# MiRNA detection in cervical exfoliated cells for missed high-grade lesions in women with LSIL/CIN1 diagnosis after colposcopy-guided biopsy

**DOI:** 10.1186/s12885-019-5311-3

**Published:** 2019-01-30

**Authors:** Jing Ye, Xiao-dong Cheng, Bei Cheng, Yi-fan Cheng, Xiao-Jing Chen, Wei-guo Lu

**Affiliations:** 1 Department of Gynecologic Oncology, Women’s Hospital, School of Medicine, Zhejiang University, Hangzhou, 310006 Zhejiang China; 2 Women’s Reproductive Health Laboratory of Zhejiang Province, Women’s Hospital, School of Medicine, Zhejiang University, Hangzhou, 310006 Zhejiang China

**Keywords:** Low-grade squamous intraepithelial lesion/cervical intraepithelial neoplasia grade 1(LSIL/CIN1), High-grade lesions, miRNA expression, Cervical exfoliated cells, Colposcopy-guided biopsy

## Abstract

**Background:**

Low-grade squamous intraepithelial lesion/cervical intraepithelial neoplasia grade 1 (LSIL/CIN1) preceded by colposcopy guided biopsy is recommended conservative follow-up, although some of these lesions are actually high-grade lesions, which are missed on an initial colposcopy. Therefore, in this work, we evaluate the potential role of miRNA detection in cervical exfoliated cells in a clinic-based population for predicting missed high-grade lesions in women diagnosed with LSIL/CIN1 after colposcopy-guided biopsy.

**Methods:**

A total number of 177 women with a diagnosis of LSIL/CIN1 obtained by colposcopy-guided biopsy were grouped into two categories according to the histology of the conization specimens: consistent LSIL/CIN1 group (surgical pathology consistent with colposcopic diagnosis) and missed high-grade lesion group (surgical pathology found high-grade lesion). The expression of eight miRNAs, such as miRNA195, miRNA424, miRNA375, miRNA218, miRNA34a, miRNA29a, miRNA16–2, and miRNA20a was detected by real time-quantitative polymerase chain reaction (RT-qPCR) in cervical exfoliated cells of the 177 patients. Pearson Chi-Square was used to compare the performance efficiency of patients’ characteristics. Nonparametric Man-Whitney U test was used to assess differences in miRNA expression. The receiver operating characteristic (ROC) curve was used to assess the performance of miRNA evaluation in detecting missed high-grade lesions.

**Results:**

Among the 177 women with biopsy-confirmed CIN1, 15.3% (27/177) had CIN2+ in the conization specimen (missed high-grade lesion group) and 84.7% (150/177) had CIN1-(consistent LSIL/CIN1 group). The relative expression of miRNA-195 and miRNA-29a in the missed high-grade lesion group was significantly lower than that in the consistent LSIL/CIN1 group. The relative expression of miRNA16–2 and miRNA20a in the missed high-grade lesion group was significantly higher than that in the consistent LSIL/CIN1 group. No significant difference was observed between these two groups regarding the other four miRNAs. Of these significant miRNAs, miRNA29a detection achieved the highest Youden index (0.733), sensitivity (92.6%), positive predictive value (46.2%), negative predictive value (98.3%) and higher specificity (80.7%) when identifying missed high-grade lesions.

**Conclusions:**

Detection of miRNA might provide a new triage for identifying a group at higher risk of missed high-grade lesions in women with colposcopy diagnosis of LSIL/CIN1.

**Electronic supplementary material:**

The online version of this article (10.1186/s12885-019-5311-3) contains supplementary material, which is available to authorized users.

## Background

Cervical cancer is the fourth most common malignancy in women worldwide, which account for 528,000 new cases and 266,000 mortalities each year [1], while it is the second most commonly diagnosed cancer in developing countries [2]. Ninety-nine percent of cervical cancer cases are caused by persistent infections with high risk human papillomavirus (HR-HPV) [3]. However, the development into invasive cervical cancer from HR-HPV infection takes a long time. Therefore implementation of screening programs with HPV and Pap test are recommended for an early detection of cervical precancerous lesions, and colposcopy should be developed for a more detailed examination [2].

With the promotion of cervical cancer screening, more and more low grade lesions (histological low-grade squamous intraepithelial lesion -LSIL-, also termed cervical intraepithelial neoplasia grade 1 -CIN1- in the prior edition of three-tier terminology), diagnosed by colposcopy guided biopsy, are detected. A CIN1 diagnosis does not represent a significant risk factor for CIN3+ above the risk attributed to its molecular cause, genotype-specific HPV infection. CIN1 is not a target of screening and CIN1 should not be treated [4]. In follow-up of women with a negative cervical biopsy (normal/CIN1), the risk of CIN3+ was similar for women with normal biopsy and CIN1 biopsy [5]. Le et al. used CIN1 in colposcopy-guided biopsy as a startpoint. A normal histology could also be used as a startpoint because the risk of CIN3+ is the same for all women with a negative cervical biopsy [5]. After a negative cervical biopsy, a normal first follow-up cytology provided a CIN3+ risk considered acceptable to recommend return to routine screening in 3 years. Cytology and HPV co-testing in post-colposcopy follow-up of negative biopsies may improve risk stratification [6]. For women with antecedent HPV-positive/ASC-US (atypical squamous cells of undetermined significance) or LSIL, a single negative post-colposcopy co-testing reduced their risk to a level consistent with a 3-year return. For women with antecedent atypical squamous cells-cannot exclude high-grade squamous intraepithelial lesion(ASC-H)or equal or greater than high-grade squamous intraepithelial lesion(HSIL+), no single negative test result sufficed to reduce their risk to a level consistent with a 3-year return [7, 8]. The probability of progression to high grade in LSIL/CIN1 women is very low and not different from women with HPV+ and normal epithelium. CIN1 is not considered anymore a precursor of CIN3 and cancer, but a different way of reproduction of the virus. The increased risk is due to the presence of the virus that can start a new pattern of reproduction, not from the evolution of CIN1 productive infection [9, 10]. Therefore, LSIL/CIN1 diagnosed by colposcopy guided-biopsy is recommended to conservative follow-up [11, 12].

However, the accuracy and reproducibility of colposcopy-guided biopsies are limited [13, 14]. Colposcopic diagnosis accuracy mainly depends on colposcopist experience and the number of cervical biopsies performed [15]. The sensitivity of conventional colposcopy for the detection of CIN2+ is poor. In the ASCUS-LSIL Triage Study, the sensitivity for CIN2+ of an online colpophotographic assessment of high-grade disease was 39% [14]. Zuchna et al. reported 66.2% sensitivity of CIN2+ when up to three guided cervical biopsies were taken regarded as a diagnostic test with the cone specimen as reference standard [16]. Regardless of skill, performing more biopsies increases the sensitivity of colposcopy [15].

Now that colposcopic diagnosis may be part of subjectivity [17], high-grade lesions may be missed with colposcopy guided biopsy. In view of the different clinical procedures between low grade lesion (LSIL/CIN1) and high grade lesion, it is meaningful to search for a new triage to reduce missed high-grade lesions from colposcopy guided-biopsy diagnosed LSIL/CIN1.

MicroRNAs (miRNAs) are small non-coding RNA of approximately 22 nucleotides that regulate gene expression through different signaling pathways. miRNA dysregulation is associated with a variety of human malignancies, such as breast, lung, colon, and cervical cancer [18–21]. Accumulating evidences indicate that some oncogenic miRNAs are associated with cervical tumorigenesis, such as miRNA-10a, miRNA-21, miRNA-19, and miRNA-146a [22–24]. Conversely, miRNA-375, miRNA-424, and miRNA-218 are tumor suppressive miRNAs, which are down-regulated in human cervical cancer [25–27]. Some mechanisms of these dysregulated miRNAs in cervical carcinogenesis have been confirmed, such as the ones of miR-375, miR-424, and miR-218, which participate in cervical carcinogenesis via targeting Sp1, Chk1, and LAMB3, respectively [28–30], while miR-34a is involved in the HPV E6-p53 pathway [31, 32]. In clinical applications, miRNA expression profiles are promising biomarkers for the early diagnosis, classification or outcome prediction of human cancer. For example, specific miRNA, such as miRNA-122 and miRNA-192, are abundant in the liver and exhibit dose- and exposure duration-dependent changes in the plasma, suggesting the potential of using specific circulating miRNAs as sensitive and informative biomarkers for drug-induced liver injury [33].

Recently, several studies detected miRNAs in serum or plasma [34], urine [35], and saliva [36], which are all noninvasive samples for cancer diagnosis and obtained promising results. Cervical exfoliated cells as residual sample of screening can be also used as noninvasive samples for miRNA detection for cervical lesions diagnosis. The clinical value of miRNAs as markers for predicting missed high-grade lesions in colposcopy diagnosed LSIL/CIN1 has not been reported.

In this study, we selected eight previously studied dysregulated miRNAs (miRNA195, miRNA424, miRNA375, miRNA218, miRNA34a, miRNA29a, miRNA16–2 and miRNA20a) as candidate biomarkers for predicting missed high-grade lesions in colposcopy diagnosed LSIL/CIN1. miRNAs expression was detected by real time-quantitative polymerase chain reaction (RT-qPCR).

## Methods

A cross-sectional study was carried out from May 2014 to December 2016 in the Women’s Hospital, School of Medicine, Zhejiang University, China. A total number of 177 subjects diagnosed as LSIL/CIN1 by colposcopy-guided biopsy (colposcopy was adequate and endocervical curettage was negative), who refused conservative follow-up and chose surgery (conization of cervix), were recruited in this study. Women were excluded according to the following criteria: (1) cytological diagnosis is AGC (Atypical glandular cells); (2) histological flat condyloma, koilocytotic atypia, and koilocytosis; (3) history of surgically or ablatively treated cervix; (4) previously confirmed cervical cancer or its precursor, or other malignancies; (5) presence of immunosuppression; (6) pregnancy. Cervix conization was conducted within three months after the first colposcopy-guided biopsy. Subjects were grouped into two categories according to the histology of the conization specimens: consistent LSIL/CIN1 group (surgical pathology consistent with the colposcopic diagnosis) and missed high-grade lesion group (surgical pathology found high-grade lesion). All eligible subjects underwent HPV and Pap test at baseline. Twenty HR-HPV negative subjects were detected and they have been fully informed of the low risk of disease progression in cases of HR-HPV negative. However, these women still chose surgery because of fear of false-negative. Information on age, smoking, number of lifetime sexual partners, and age at first sexual intercourse were collected via an interviewer-administered structured questionnaire.

This study was approved by the Human Research Ethical Committee of the Women’s Hospital, School of Medicine, Zhejiang University, China, with protocol No. 20110014. Written informed consent was obtained from each participants included in this study.

Pap test was performed according to the 2001 Bethesda System [37]. HPV genotyping was detected by HPV GenoArray test kit (Hybribio, Hong Kong, China) according to the manufacturer’s instructions, as previously described [38, 39]. Colposcopy-guided biopsy was performed according to the standardized protocol. The histological diagnose of the colposcopy guided biopsy and conization specimens were analyzed by the same pathologists according to the Lower Anogenital Squamous Terminology (LAST) recommendations [40]. The histological consensus was reached by a group of experts in case of disagreement.

Relative expressions of miRNAs in cervical exfoliated cells was detected as previously described [41]. In brief, total RNA of cervical exfoliated cells was extracted using Trizol reagent (Invitrogen, Carlsbad, CA).Next, cDNA was synthesized from RNA. U6 was used as a stable reference gene for normalization. Primers used in miRNA detection are shown in Additional file 1: Table S1. Real-time PCR for miRNA was performed as previously reported. miRNA relative expression was calculated based on the following equation: miRNA relative expression = 2^-ΔCt^, where ΔCt = Ct (miRNA) – Ct (U6).

Statistical analysis was performed using SPSS software version 17.0 (SPSS Inc., Chicago, IL). Two-sided *P* value less than 0.05 was considered statistically significant. Socio-demographic characteristics were compared by Pearson Chi-Square test. miRNA expression comparison was performed by the nonparametric Mann–Whitney U test. The receiver operating characteristic (ROC) curve was used to assess the performance of miRNA expression for detecting missed high-grade lesions. Pearson Chi-Square was used to compare performance efficiency between Pap test and miRNA detection. The optimal cutoff value of each miRNA was determined by the maximal Youden index [42]. For the Pap test, ASC-H+ (equal or greater than atypical squamous cells-cannot exclude high-grade squamous intraepithelial lesion) was used as the cutoff value. Two histological cutoffs were used: CIN1- and CIN2 + .

## Results

A total of 177 women with LSIL/CIN1 diagnosis obtained by colposcopy-guided biopsy and treated by conization of cervix within three months were included in the study. Among them, 15.3% (27/177) of the women with CIN1 in biopsy had CIN2+ in the conization specimen and 84.7% (150/177) had CIN1-.

Table 1 shows the distribution of the baseline characteristics of the subjects. Cervical HR-HPV infection was found in 86.7% of consistent LSIL/CIN1 group and 100% of missed high-grade lesion group, and the difference was statistically significant. ASC-H+ was found in 8.7% of consistent LSIL/CIN1 group and 22.2% of missed high-grade lesion group, and the difference was also statistically significant. The difference between the two groups was not significant regarding age, smoking, number of lifetime sexual partners and the age at first sexual intercourse.

**Table 1:** Characteristics of the study subjects

	Histology		*P* value
	Consistent LSIL/CIN1 group (*n* = 150)	Missed high-grade lesion group (*n* = 27)	
Age (years)			
Mean (SD)	39.4 (±6.6)	39.8 (±7.1)	
< 40	68 (45.33%)	12 (44.44%)	0.932
≥ 40	82 (54.67%)	15 (55.56%)	
HR-HPV			
Positive	130 (86.67%)	27 (100%)	0.044
Negative	20 (13.33%)	0 (0%)	
HPV genotyping			
Positive of HPV16,18	35 (23.33%)	8 (29.63%)	0.483
Negative of HPV16,18	115 (76.67%)	19 (70.37%)	
Pap test			
ASC-H-	137 (91.33%)	21 (77.78%)	0.036
ASC-H+	13 (8.67%)	6 (22.22%)	
Smoking			
Non-smoker	105 (70%)	21 (77.78%)	0.411
Smoker*	45 (30%)	6 (22.22%)	
Number of lifetime sexual partners			
1	123 (82%)	21 (77.78%)	0.604
2+	27 (18%)	6 (22.22%)	
Age at first sexual intercourse (years)			
< 20	40 (26.67%)	7 (25.93%)	0.474
20–24	90 (60%)	14 (51.85%)	
> 24	20 (13.33%)	6 (22.22%)	

The *P* value was calculated by the Pearson Chi-Square test

LSIL/CIN1: Low-grade squamous intraepithelial lesion/cervical intraepithelial neoplasia grade 1

SD Standard Deviation

HPV Human papillomavirus

HR-HPV High-risk human papillomavirus

ASC-H+ equal or greater than atypical squamous cells-cannot exclude high-grade squamous intraepithelial lesion

*including smokers and second-hand smoker subjects

Table 2 shows the relative expression of the eight candidate miRNAs in cervical exfoliated cells between consistent LSIL/CIN1 group and missed high-grade lesion group. The relative expression of miRNA-195 and miRNA-29a in the missed high-grade lesion group was significantly lower than that in the consistent LSIL/CIN1 group. Conversely, the relative expression of miRNA16–2 and miRNA20a in the missed high-grade lesion group was significantly higher than that in the consistent LSIL/CIN1 group. No significant difference between these two groups was observed regarding miR-424, miR-375, miR-218 and miR-34a expression.

**Table 2:** Comparison of miRNA expression in cervical exfoliated cells between consistent LSIL/CIN1 group and missed high-grade lesion group

Variable	Consistent LSIL/CIN1 group (*n* = 150)Median (IQR)	Missed high-grade lesion group (*n* = 27)Median (IQR)	*p*
miRNA195 (×10^−5^)	1.81(1.30–2.25)	0.72(0.53–0.96)	***< 0.001***
miRNA424 (×10^−5^)	1.50(0.48–3.87)	1.08(0.55–2.98)	0.712
miRNA375 (×10^−3^)	6.67(3.39–13.66)	4.27(2.82–16.66)	0.547
miRNA218 (×10^−5^)	2.60(1.14–6.34)	2.51(1.05–9.17)	0.775
miRNA34a (×10^−4^)	4.38(1.69–9.48)	3.58(1.64–10.10)	0.664
miRNA29a (×10^−5^)	1.975(1.46–2.59)	0.86(0.63–1.03)	***< 0.001***
miRNA16–2(× 10^−4^)	0.494(0.38–0.60)	0.638(0.54–0.73)	***< 0.001***
miRNA20a (× 10^− 4^)	0.5005(0.33–0.68)	0.861(0.74–0.98)	***< 0.001***

LSIL/CIN1: Low-grade squamous intraepithelial lesion/cervical intraepithelial neoplasia grade 1

IQR Interquartile range. *P* value in boldface is less than 0.05 and considered statistically significant

**Fig. 1:**
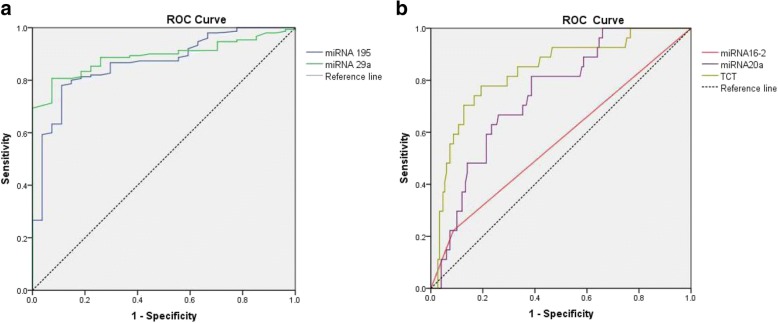
Receiver operating characteristic (ROC) curves of miRNA detection and Pap test in identifying missed high-grade lesions from colposcopy-guided biopsy diagnosed LSIL/CIN1. **a** ROC curves of down-regulated miRNAs (miRNA195 and miRNA29a) (compared with consistent LSIL/CIN1 group); **b** ROC curves of up-regulated miRNAs (miRNA16–2 and miRNA20a) (compared with consistent LSIL/CIN1 group) LSIL/CIN1: Low-grade squamous intraepithelial lesion/cervical intraepithelial neoplasia grade 1

Furthermore, we plotted the ROC curves for miRNA195, miRNA29a, miRNA16–2, miRNA20a and Pap test (Fig. 1). Table 3 shows the performance parameters of miRNA detection and Pap test for identifying missed high-grade lesions from colposcopy guided biopsy diagnosed LSIL/CIN1. To identify missed high-grade lesions, four miRNAs (miRNA-195, miRNA-20a, miRNA16–2, and miRNA-29a) achieved an area under curve (AUC) above 0.74, suggesting clinical significance. Compared with the Pap test, miRNA detection achieved a significantly higher sensitivity, negative predictive value, and compared positive predictive value, but significantly lower specificity. The maximal Youden index of miRNA195, miRNA29a, miRNA16–2, miRNA20a and Pap test were 0.669, 0.733, 0.428, 0.585 and 0.135, respectively, suggesting better performance of miRNA detection in identifying missed high-grade lesions than Pap test.

**Table 3:** Comparison of performance efficiency between miRNA detection and Pap test for identifying missed high-grade lesions from colposcopy guided biopsy diagnosed LSIL/CIN1

miRNA	AUC (95% CI)	Sensitivity		Specificity		Positive predictive value		Negative predictive value	
		95% CI	*P*	95% CI	*P*	95% CI	*P*	95% CI	*P*
miRNA195	0.86 (0.79–0.93)	88.9(69.7–97.0)	***< 0.001***	78(70.3–80.1)	***0.0013***	42.1(29.4–55.8)	0.41	97.5(92.3–99.3)	***0.0015***
miRNA29a	0.89 (0.84–0.93)	92.6(74.2–98.7)	***< 0.001***	80.7(73.2–86.4)	***0.0077***	46.2(32.8–60.2)	0.26	98.3(93.6–99.7)	***0.00040***
miRNA16–2	0.74 (0.65–0.83)	81.5(61.2–92.9)	***< 0.001***	61.3(53.0–69.0)	***< 0.001***	27.5(18.3–38.8)	0.71	94.8(87.8–98.0)	***0.037***
miRNA20a	0.83 (0.74–0.91)	77.8(57.2–90.6)	***< 0.001***	80.7(73.2–86.4)	***0.0077***	42(28.4–56.7)	0.42	95.2(89.5–98.0)	***0.014***
pap test	0.56(0.44–0.69)	22.22(9.3–42.7)		91.3(85.3–95.1)		31.5(13.5–56.5)		86.7(80.1–90.3)	

*P* value refers to the comparison between miRNA and Pap test with Pearson Chi-square test. All statistical tests were two-sided. *P* value in boldface is less than 0.05 and considered statistically significant

The 95% confidence interval (CI) of proportions was calculated according to the following equation: p ±$\sqrt{1.96\mathrm{p}\left(1-p\right)/n}$, where n is the case number involved in the calculation of the proportion

AUC Area under curve

CI Confidence interval

According to the maximal Youden index, 1.241 × 10^− 5^, 1.325 × 10^− 5^, 0.542 × 10^− 4^, and 0.732 × 10^− 4^ were identified as the cutoff values of miRNA-195, miRNA-29a, miRNA16–2 and miRNA-20a, respectively, for missed high-grade lesions identification. Of these four miRNAs, miRNA-29a detection achieved the greatest AUC (0.89) with the highest Youden index (0.733), sensitivity (92.6%), positive predictive value (46.2%), negative predictive value (98.3%) and higher specificity (80.7%) when identifying missed high-grade lesions.

## Discussion

This study evaluated whether miRNA expression in cervical exfoliated cells might be associated with missed high-grade lesions in subjects with a diagnosis of LSIL/CIN1 after colposcopy-guided biopsy. The results showed that 15.3% (27/177) LSIL/CIN1 diagnosed by colposcopy-guided biopsy were missed high-grade lesions, suggesting that colposcopy-guided biopsy samples are not always representative of the severity of the cervical lesions [43]. In a Norwegian study of 520 women with a negative cervical biopsy (normal/CIN1), 124 women (23.8%) had CIN2+ in follow-up biopsy including seven cases of invasive cervical cancer [5]. Boonlikit et al. reported an agreement rate of 66.0% between histological diagnosis of the biopsy and conization specimens (Kappa = 0.2; fair agreement) in women under 50 years [44]. Zuchna et al. reported 66.2% sensitivity of CIN2+ when up to three guided cervical biopsies were taken regarded as a diagnostic test with the cone specimen as reference standard [17]. The biopsy failure rate seems to increase with the low severity of the histological diagnosis [45]. The reason for the missed diagnosis of colposcopy-guided biopsy may be associated with the experience of colposcopists in grading cervical lesion and the number of cervical biopsies performed [15, 46, 47]. Indeed, performing more biopsies increases the sensitivity of colposcopy regardless of skills [15]. According to the different clinical principles between low grade lesion (LSIL/CIN1) and high grade lesion, it is necessary to find new detection methods to decrease missed high-grade lesions from LSIL/CIN1 diagnosed by colposcopy-guided biopsies.

Correlational studies are limited. It has been reported that cervical cancer risk factors (such as HR-HPV infection, smoking and high sexual activity) are higher in women with high-grade lesions [48–50]. Our study reported 15.3% missed high-grade lesions in women with initial diagnosis of LSIL/CIN1 by colposcopy-guided biopsies. In this study, no significant association was found in age, smoking, number of lifetime sexual partners, and age at first sexual intercourse, while HR-HPV infection and Pap test grading were significantly associated with missed high-grade lesions. No missed lesion in HR-HPV negative women was found. Although the *P* value for this observation was close to 0.05, the direction and the strength of the association were notable. The *P* value is affected by many factors, among them the sample size is an important one. The *p*-value close to 0.05 is due to small sample size of our study. Further large sample research is needed to validate the observation.

Pap test grading was associated with missed high-grade lesions in our study. According to the ASCCP guideline, women with ASC-H+ were recommended for conservative treatment or excisional procedure, while women with Pap test less serious than ASC-H were recommended for conservative treatment only [9]. Therefore, in our study, Pap test diagnosis was compared with the miRNAs expression for detecting missed high-grade lesions. Compared with previous literature data, the accuracy of our Pap test is slightly lower than that observed in cohort studies in which the gold standard was determined through follow up [51, 52]. This could be due to ascertainment biases in follow up studies or to lower progression of high grade lesion found in low grade cytology than those in high grade cytology or to low sensitivity of local cytology (at least in the distinction between low and high grade).

miRNA expression in cervical exfoliated cells was analyzed to find the correlation between gene expression and missed high-grade lesions in colposcopy-guided biopsy. Cervical exfoliated cells were ideal samples for cervical cancer screening, which have been widely used in HPV testing and Pap test [53, 54]. miRNA195, miRNA424, miRNA375, miRNA218, miRNA34a, miRNA29a, miRNA16–2 and miRNA20a have been validated associated with transition from normal cervix to both precancerous stages (atypical dysplasia) and cancer [28, 31, 41, 55]. Consistently, miRNA-195 and miRNA-29a expression in cervical exfoliated cells was significantly lower in missed high-grade lesion group compared with consistent LSIL/CIN1 group, while miRNA16–2 and miRNA20a expression was significantly higher in women with missed high-grade lesions in our present study.

In this study, according to the higher AUC and Youden index, miRNA detection showed better effectiveness in identifying missed high-grade lesions compared with Pap test. Of these four miRNAs, miRNA-29a showed the greatest AUC with the highest Youden index, sensitivity, positive predictive value, negative predictive value and higher specificity in identifying missed high-grade lesions. MiRNA-29a was significantly down-regulated in several types of human cancer (including prostate cancer, pancreatic cancer, and lung adenocarcinoma), suggesting that it acts as a putative tumor-suppressor miRNA [56–58]. Down regulation of miRNA-29a resulted in HSP47 overexpression, which was associated with transition from normal cervix to both precancerous stages (atypical dysplasia) and cancer [59, 60]. Our results is consistent with previous studies, and suggest that miRNA-29a in cervical exfoliated cells could be used as a candidate biomarker in predicting missed high-grade lesions in women with LSIL/CIN1 diagnosed by colposcopy-guided biopsy.

The main limit of this study is that we do not really know the clinical significance of these high grade lesions missed by colposcopy-guided biopsy. Studies with random biopsy or studies like us showed high proportion of missed CIN2+, while careful follow up of women with LSIL/CIN1 usually found very low incidence of CIN2+ in this group [5]. There are several insights that these lesions could be highly regressive [61, 62]. It is now clear from follow up studies that the probability of having a CIN2+ in the two years after a colposcopy is almost identical in women HPV+ with negative colposcopy and negative cytology than those with LSIL/CIN1 [5]. Maybe this triage test can be used to modulate the re-testing interval for follow up in all HPV+ women, other than identifying women to be treated. The positive predictive value suggests that this application could avoid relevant overtreatment.

## Conclusion

Our study suggests a potential application of miRNA detection in cervical exfoliated cells. Our findings from a clinic-based population demonstrated that the detection of miRNA-29a in subjects with colposcopy-guided biopsy diagnosed LSIL/CIN1represents a promising marker for detecting missed high-grade lesions. miRNA detection might provide an additional option for triage of colposcopy-guided biopsy diagnosed LSIL/CIN1. Further research in a general population is needed to validate these findings.

## Additional file

Additional file 1: **Table S1.** Primes sequence of the miRNA. (XLS 21 kb)

## Abbreviations

ASC-H+: Equal or greater than atypical squamous cells-cannot exclude high-grade squamous intraepithelial lesion
AUC: Area Under Curve
CI: Confidence interval
HPV: Human papillomavirus
HR-HPV: High-risk Human papillomavirus
IQR: Interquartile range
LSIL/CIN1: Low-grade squamous intraepithelial lesion/cervical intraepithelial neoplasia grade 1
SD: Standard Deviation
